# Deciphering Pharmacological Mechanism of Buyang Huanwu Decoction for Spinal Cord Injury by Network Pharmacology Approach

**DOI:** 10.1155/2021/9921534

**Published:** 2021-04-22

**Authors:** Zhencheng Xiong, Feng Yang, Wenhao Li, Xiangsheng Tang, Haoni Ma, Ping Yi

**Affiliations:** ^1^Department of Spine Surgery, China-Japan Friendship Hospital, Beijing 100029, China; ^2^Beijing University of Chinese Medicine, Beijing 100029, China

## Abstract

**Objective:**

The purpose of this study was to investigate the mechanism of action of the Chinese herbal formula Buyang Huanwu Decoction (BYHWD), which is commonly used to treat nerve injuries, in the treatment of spinal cord injury (SCI) using a network pharmacology method.

**Methods:**

BYHWD-related targets were obtained by mining the TCMSP and BATMAN-TCM databases, and SCI-related targets were obtained by mining the DisGeNET, TTD, CTD, GeneCards, and MalaCards databases. The overlapping targets of the abovementioned targets may be potential therapeutic targets for BYHWD anti-SCI. Subsequently, we performed protein-protein interaction (PPI) analysis, screened the hub genes using Cytoscape software, performed Gene Ontology (GO) annotation and KEGG pathway enrichment analysis, and finally achieved molecular docking between the hub proteins and key active compounds.

**Results:**

The 189 potential therapeutic targets for BYHWD anti-SCI were overlapping targets of 744 BYHWD-related targets and 923 SCI-related targets. The top 10 genes obtained subsequently included AKT1, IL6, MAPK1, TNF, TP53, VEGFA, CASP3, ALB, MAPK8, and JUN. Fifteen signaling pathways were also screened out after enrichment analysis and literature search. The results of molecular docking of key active compounds and hub target proteins showed a good binding affinity for both.

**Conclusion:**

This study shows that BYHWD anti-SCI is characterized by a multicomponent, multitarget, and multipathway synergy and provides new insights to explore the specific mechanisms of BYHWD against SCI.

## 1. Introduction

Spinal cord injury (SCI) is a group of disorders in which the integrity and continuity of the spinal cord are disrupted by trauma, tumor, hypoxia, inflammation, and other factors [1]. According to the World Health Organization (WHO) report, about 250,000 to 500,000 people worldwide suffer from SCI every year, and the population is mainly under 30 years old [2]. The Beijing SCI Epidemiology Survey Group reported in 2002 that the incidence of SCI in Beijing was about 60/10^6^, an increase of nearly 10 times compared to the late 1980s, and that the per capita hospitalization cost was 27819.3 CNY [3]. The high disability rate and high consumption of SCI have imposed a heavy burden on the families of patients and society, while how to protect and restore injured neurons after SCI has been considered a great challenge in clinical and experimental research [4]. There is still a lack of effective treatment for SCI, but it is known that traditional Chinese medicine (TCM) has a long history and rich practical experience, which may be explored for the treatment of SCI [2].

Modern Chinese medicine scholars classify SCI as “Ti Duo” and “Wei Zheng” based on the TCM classics “Ling Shu Jing-Han Re Bing” and “Su Wen-Wei Lun” [3]. Based on the analysis of the etiology and pathogenesis of SCI by TCM, as well as the pathological changes of the damaged Governor Vessel and the symptoms corresponding to SCI, the main treatment of TCM is to unblock Governor Vessel and strengthen “Yang Qi,” supplemented by tonifying the kidney and filling the essence, strengthening the tendons and bones, activating blood circulation and removing blood stasis, and warming the meridians [3]. Buyang Huanwu Decoction (BYHWD), recorded in “Yi Lin Gai Cuo” by Wang Qingren in the Qing Dynasty, has been used in China for hundreds of years to treat stroke-related disabilities and improve the recovery of neurological function [5]. The whole formula of BYHWD consists of seven herbs: Chishao (*Radix Paeoniae Rubra*), Chuanxiong (*Rhizoma Chuanxiong*), Danggui (*Radix Angelicae Sinensis*), Dilong (*Pheretima*), Huangqi (*Radix Astragali*), Honghua (*Flos Carthami*), and Taoren (*Semen Persicae*) [5]. Studies have shown that BYHWD may promote the repair of SCI by upregulating the expression of Notch1 gene [6], and BYHWD combined with bone marrow mesenchymal stem cells (BMSCs) transplantation can promote the recovery after SCI by saving axotomized red nucleus neurons in rats [4]. Although some progress has been made in the study of BYHWD for SCI, the complexity of herbal prescriptions with multiple components, targets, and pathways makes the study of the specific mechanism of action of BYHWD for SCI difficult to clarify.

**Figure 1:**
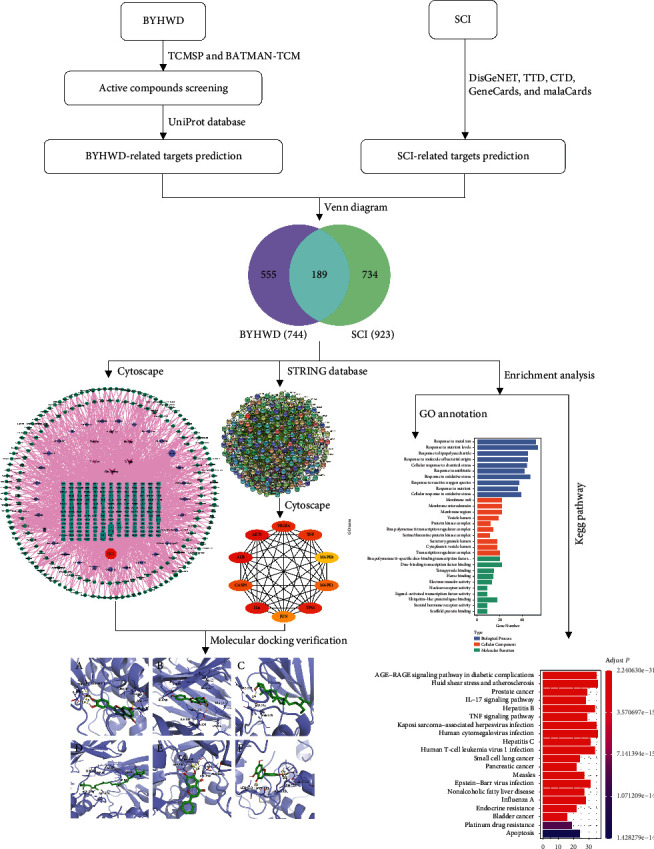
The overall flowchart of this study.

In the last decade, with the development of modern multiomics theories such as genomics, proteomics, and metabolomics, the introduction of a systems biology perspective, and the application of bioinformatics, the concept of network pharmacology has emerged. According to the network basis of drug action, network pharmacology methods, including protein-protein interaction (PPI) network, Gene Ontology (GO) annotation, and Kyoto Encyclopedia of Genes and Genomes (KEGG) pathway enrichment analysis, are applied to infer the specific pathways of multimolecular drug action in the human body, which is consistent with the multicomponent, multitarget, and multipathway synergy of Chinese herbal medicine and helps to clarify the mechanism of action of BYHWD (Figure 1).

## 2. Materials and Methods

### 2.1. Acquisition of BYHWD-Related Targets and SCI-Related Targets

The compounds related to BYHWD were obtained by searching “Chishao or CHI SHAO or *Radix Paeoniae Rubra*,” “Chuanxiong or CHUAN XIONG or *Rhizoma Chuanxiong*,” “Danggui or DANG GUI or *Radix Angelicae Sinensis*,” “Dilong or DI LONG or *Pheretima*,” “Huangqi or HUANG QI or *Radix Astragali*,” “Honghua or HONG HUA or *Flos Carthami*,” and “Taoren or TAO REN or *Semen Persicae*” in Traditional Chinese Medicine Systems Pharmacology (TCMSP, version 2.3, https://tcmspw.com/tcmsp.php) database [7], and then the active compounds that met the criteria (oral bioavailability (OB) ≥ 30 and drug-likeness (DL) ≥ 0.18) were further screened by the absorption, distribution, metabolism, and excretion (ADME) principle [8, 9], and the corresponding target protein information of the active compounds was also obtained. Subsequently, by using the UniProt database (https://www.uniprot.org/) (species should be set to “*Homo sapiens*”), the target protein name is converted into the corresponding gene name and UniProt ID [10]. The compounds and targets related to BYHWD were also obtained directly by searching the above keywords in the BATMAN-TCM platform (http://bionet.ncpsb.org/batman-tcm/) according to the set criteria (score cutoff >25, adjusted *P* value < 0.05) [11]. Finally, the results of the two databases are summarized, integrated, and deduplicated to obtain BYHWD-related targets.

DisGeNet database (https://www.disgenet.org/, v7.0) (score ≥ 0.01) [12], GeneCards (https://www.genecards.org/) (relevance score ≥ 20) [13], MalaCards (https://www.malacards.org/) [14], Comparative Toxicogenomics Database (CTD) (http://ctdbase.org/, last update by June 2020) (inference score ≥ 20) [15], and Therapeutic Target Database (TTD) (http://db.idrblab.net/ttd/, last update by June 1, 2020) [16] were used to screen out potential targets related to SCI by searching for the keyword “Spinal cord injury or Spinal cord diseases.” The results of the five databases are summarized, integrated, and deduplicated to obtain SCI-related targets.

### 2.2. Acquisition of Potential Therapeutic Targets for BYHWD Anti-SCI

Potential therapeutic targets of BYHWD in the treatment of SCI should be derived from the overlapping targets of BYHWD-related targets and SCI-related targets obtained from the Venn online tool (http://www.bioinformatics.com.cn/). Subsequently, we used Cytoscape software (version 3.7.2) to construct a network diagram of “BYHWD-active compounds-target genes-SCI” [17].

### 2.3. Construction of PPI Network and Acquisition of Hub Genes

PPI is composed of proteins that interact with each other to participate in various aspects of life processes such as biological signaling, regulation of gene expression, energy and material metabolism, and cell cycle regulation [18]. The potential therapeutic targets of BYHWD against SCI were analyzed by a PPI network using the STRING database (http://string-db.org/; version 11) with the species set to “*Homo sapiens*” and a confidence level greater than 0.4 [19]. Subsequently, the PPI network was visualized by Cytoscape software (version 3.7.2).

To obtain the hub genes for BYHWD anti-SCI, we use the CytoHubba plug-in in Cytoscape software, which provides 12 topological analysis methods, including Degree, Maximal Clique Centrality (MCC), BottleNeck, Betweenness, ClusteringCoefficient, Closeness, Density of Maximum Neighborhood Component (DMNC), EcCentricity, Edge Percolated Component (EPC), Maximum Neighborhood Component (MNC), Radiality, and Stress [20, 21].

### 2.4. GO and KEGG Pathway Enrichment Analysis of Potential Therapeutic Genes for BYHWD Anti-SCI

R is commonly used for statistical analysis and plotting software, and its open-source and free features have been favored by many programming enthusiasts, resulting in the birth of many convenient and efficient packages, such as the ClusterProfiler package for GO annotation and KEGG pathway enrichment analysis [22–24]. In this study, we used ClusterProfiler package (adjusted *P* value < 0.05) to explore the biological processes (BP), cellular components (CC), molecular functions (MF), and signaling pathways associated with BYHWD anti-SCI.

### 2.5. Molecular Docking of Hub Genes and Key Active Compounds

Based on the previously mined BYHWD-related targets and related active compounds, the active compounds and herbs corresponding to the top 10 hub genes need to be sorted out. The interrelationship between “herbs in BYHWD-active compounds-top 10 hub genes” was visualized by a Sankey diagram (http://sankeymatic.com/). In the following, we will select key active compounds as small molecule ligands and corresponding large molecule receptors (proteins encoded by the top 10 hub genes) for molecular docking analysis. The raw files of key active compounds (MOL2 format) can be downloaded from PubChem database (https://pubchem.ncbi.nlm.nih.gov/), and the raw files of the top 10 core genes (PDB format) can be downloaded from the RCSB protein data (http://www.rcsb.org/), and after corresponding processing by AutoDock Tool [25], they are finally converted to PDBQT format for molecular docking in Pymol software (https://pymol.org/2/; version 2.4.1) [26]. The molecular docking score reflects the binding affinity, with smaller values representing higher affinity for the binding of small molecule ligands and large molecule receptors [27].

## 3. Results

### 3.1. Acquisition Results of BYHWD-Related Targets and SCI-Related Targets

A total of 775 compounds (BATMAN-TCM: 219; after deduplication: 189) in BYHWD were preliminarily retrieved from the TCMSP database and BATMAN-TCM platform (score cutoff >25; adjusted *P* value < 0.05), of which 119 (BATMAN-TCM: 8) were from Chishao, 189 (87) were from Chuanxiong, 125 (77) were from Danggui, 87 (23) were from Huangqi, 189 (22) were from Honghua, and 66 (2) were from Taoren. Relevant compounds and targets' information for Dilong was not retrieved in either database. After setting the ADME criteria (OB ≥ 30% and DL ≥ 0.18), a total of 103 active compounds (after removing duplicates and nontarget compounds: 60) were screened from the TCMSP database, of which 29 (After removing nontarget compounds: 14) were from Chishao, 7 (6) were from Chuanxiong, 2 (2) were from Danggui, 20 (17) were from Huangqi, 22 (17) were from Honghua, and 23 (19) were from Taoren. The BATMAN-TCM platform provides the gene names directly, while the TCMSP database first provides the target protein names of the active compounds and therefore needs to be converted to gene names via the UniProt database. Finally, after integrating the results of the two databases, we obtained 244 BYHWD-related active compounds and 744 BYHWD-related targets.

After deduplication, a total of 923 SCI-related targets were obtained by mining the DisGeNET (number: 84), TTD (number: 5), CTD (number: 553), GeneCards (number: 432), and MalaCards (number: 19) databases.

### 3.2. Acquisition Results of Potential Therapeutic Targets for BYHWD Anti-SCI

**Figure 2:**
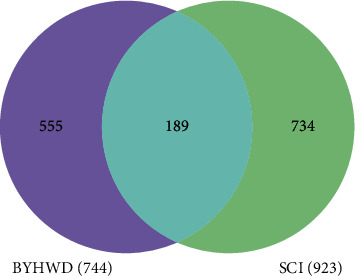
Venn diagram for BYHWD-related targets and SCI-related targets.

**Figure 3:**
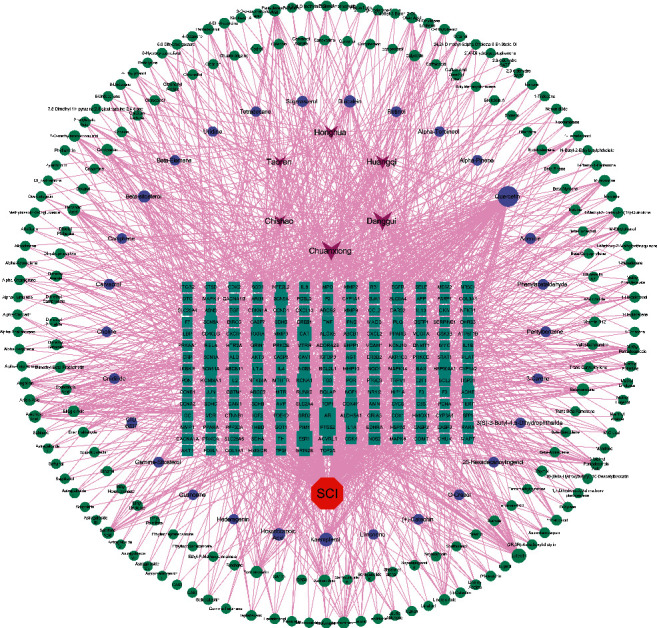
BYHWD-active compounds-target genes-SCI” network. The purple diamond represents the herbs contained in BYHWD; the red octagon represents SCI; the cyan rectangle represents the potential therapeutic targets for BYHWD anti-SCI; the light green ellipse represents the active compounds contained in BYHWD; the blue ellipse represents the common active compounds of the herb.

The overlapping targets of BYHWD-related targets and SCI-related targets were considered as potential therapeutic targets for BYHWD anti-SCI. As shown in Figure 2, we screened the overlapping targets of both by constructing a Venn diagram, and a total of 189 targets were obtained. Through multiple databases mining and construction of the Venn diagram, we obtained BYHWD-related compounds and potential therapeutic targets for BYHWD anti-SCI and finally used Cytoscape software to construct a “BYHWD-active compounds-target genes-SCI” network (Figure 3). In Figure 3, the lines between two nodes represent the existence of mutual relationships, and the larger the node is, the more relationships exist.

### 3.3. Acquisition Results of Hub Genes for BYHWD Anti-SCI and PPI Network Construction

**Figure 4:**
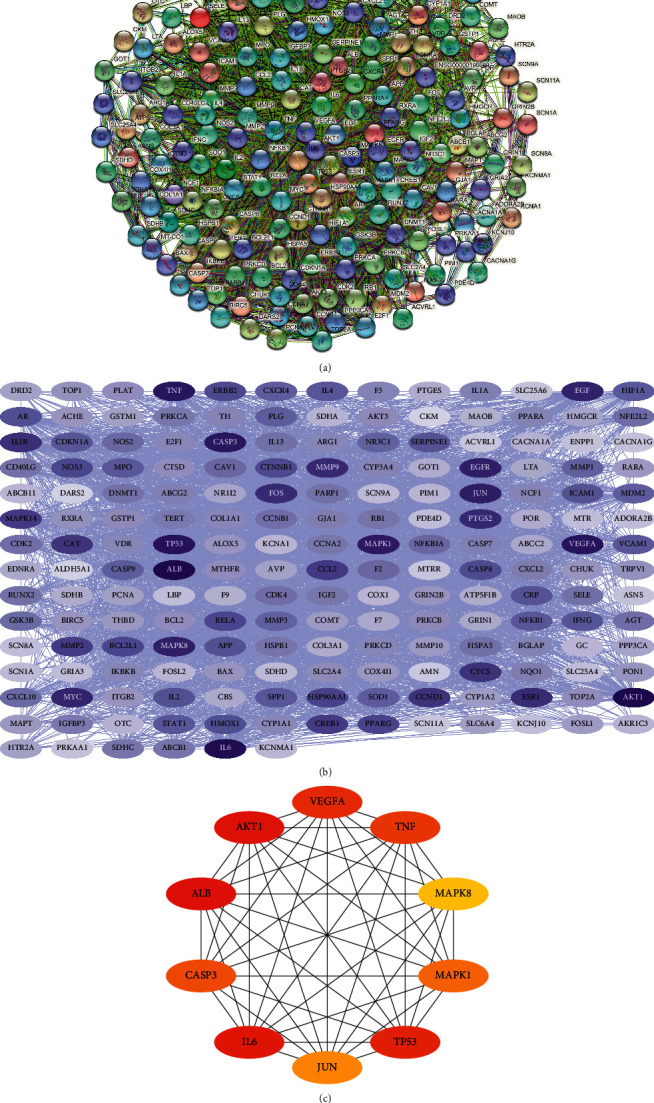
PPI network and top 10 hub genes for BYHWD anti-SCI. (a) PPI network constructed with STRING. (b) PPI network constructed with Cytoscape software (the darker the node color, the higher the number of connected proteins). (c) Top 10 hub genes for BYHWD anti-SCI were obtained by using the Degree algorithm.

In the STRING database, we first set the species to “*Homo sapiens*” and then entered 189 potential therapeutic targets for BYHWD anti-SCI to obtain the PPI network (Figure 4(a)), which involved 189 nodes and 3717 edges. The obtained TSV files were then imported into Cytoscape software (version 3.7.2) for further analysis and visualization (Figure 4(b)). To obtain the hub genes for BYHWD anti-SCI, based on the above the PPI network, we used the CytoHubba plug-in of Cytoscape software, which currently contains 12 topological analysis methods. We targeted the top 10 hub genes for BYHWD anti-SCI, and the results of the 12 algorithms each contained 10 genes. We integrated and analyzed the results to obtain a total of 45 different genes, which were then sorted by the number of algorithms to which they were attributed.  Table 1 shows the basic information on hub genes for BYHWD anti-SCI. The top 10 hub genes (AKT1, IL6, MAPK1, TNF, TP53, VEGFA, CASP3, ALB, MAPK8, and JUN) sorted are completely consistent with some algorithms (Degree, Closeness, and MNC) (Figure 4(c)).

### 3.4. Results of GO and KEGG Pathway Enrichment Analysis

**Figure 5:**
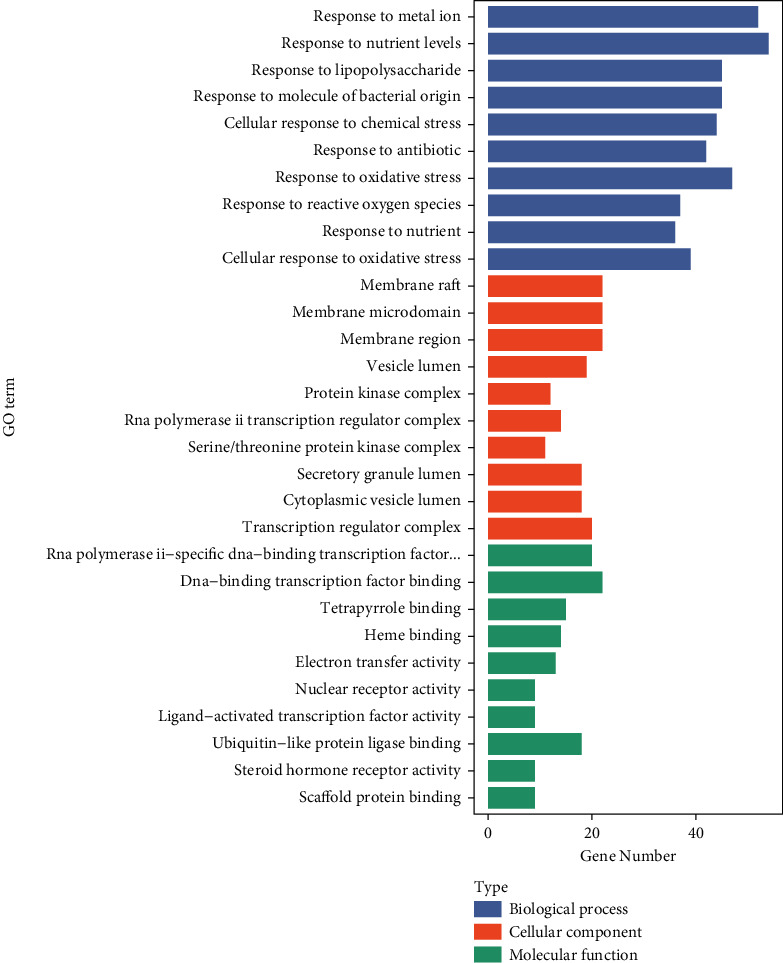
The bar chart of top 10 GO (BP, CC, and MF) enriched items.

GO and KEGG pathway enrichment analysis are important tools to explore the biological processes and signal pathways involved in clinical drug therapy for diseases, as well as key genes. Through the ClusterProfiler package in R, we performed GO enrichment analysis on the 189 potential therapeutic targets for BYHWD anti-SCI and obtained 2842 GO items (after adjustment, *P* < 0.05), including 2567 BP items, 99 CC items, and 176 MF items. We combined the top 10 enrichment results for each of GO-BP, GO-CC, and GO-MF by a bar graph (Figure 5). The basic information of the top 10 GO enrichment items is shown in Table 2.

**Figure 6:**
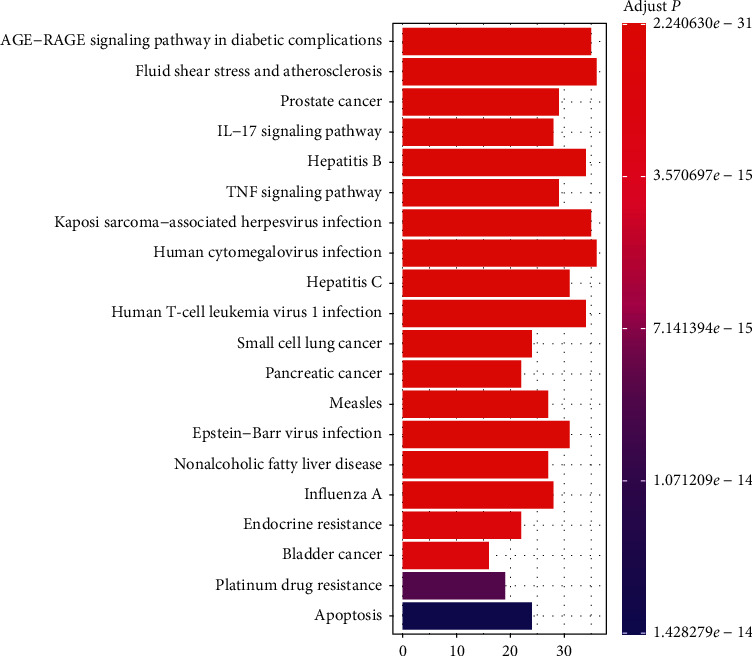
The bar chart of top 20 KEGG enriched pathways.

**Figure 7:**
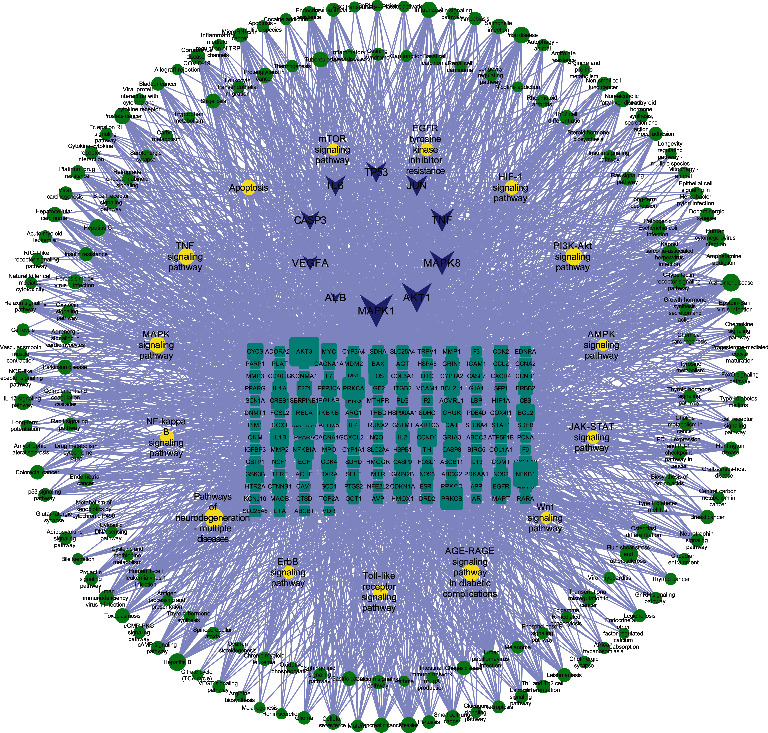
Potential therapeutic targets-pathways” network. The blue diamond represents the top 10 hub genes; the cyan rectangle represents the potential therapeutic targets for BYHWD anti-SCI; the yellow ellipse represents potential SCI-related pathways supported by literature; the green ellipse represents possible pathways related to SCI.

In the same way, through the ClusterProfiler package in R, we performed KEGG pathway enrichment analysis on the 189 potential therapeutic targets for BYHWD anti-SCI and obtained 177 KEGG pathways (after adjustment, *P* < 0.05). The top 20 KEGG enrichment pathways are listed below, including AGE-RAGE signaling pathway in diabetic complications (hsa04933), Fluid shear stress and atherosclerosis (hsa05418), Prostate cancer (hsa05215), IL-17 signaling pathway (hsa04657), Hepatitis B (hsa05161), TNF signaling pathway (hsa04668), Kaposi sarcoma-associated herpesvirus infection (hsa05167), Human cytomegalovirus infection (hsa05163), Hepatitis C (hsa05160), Human T-cell leukemia virus 1 infection (hsa05166), Small cell lung cancer (hsa05222), Pancreatic cancer (hsa05212), Measles (hsa05162), Epstein-Barr virus infection (hsa05169), Nonalcoholic fatty liver disease (hsa04932), Influenza A (hsa05164), Endocrine resistance (hsa01522), Bladder cancer (hsa05219), Platinum drug resistance (hsa01524), and Apoptosis (hsa04210). The visualization of the abovementioned KEGG enrichment pathway is realized in Figure 6. More KEGG pathways were enriched, and another fine screening of the pathways was required. Therefore, the SCI-related literature was searched in the PubMed database, and then the retrieved potentially relevant pathways were compared with the enriched 177 pathways, and finally, 15 relatively relevant pathways for SCI were obtained. Subsequently, we used Cytoscape software to visualize the network relationship between potential therapeutic targets for BYHWD anti-SCI and pathways (Figure 7).  Table 3 shows the basic information of 15 pathways that may be relevant for SCI.

### 3.5. Results of Molecular Docking of Hub Genes and Key Active Compounds

**Figure 8:**
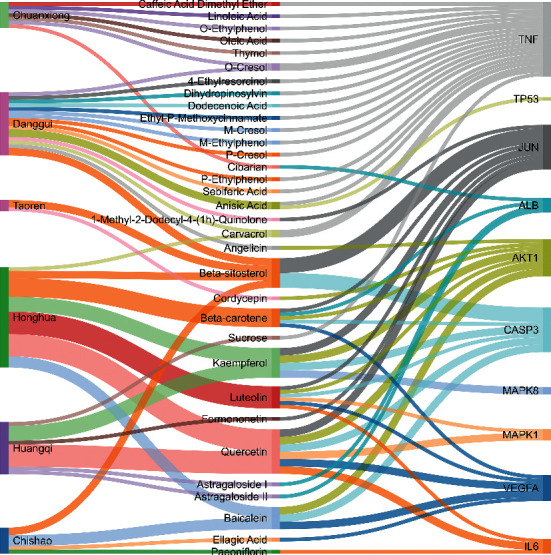
Sankey diagram of “herbs-active compounds-hub genes.” The band between the two bars represents a targeting relationship.

**Figure 9:**
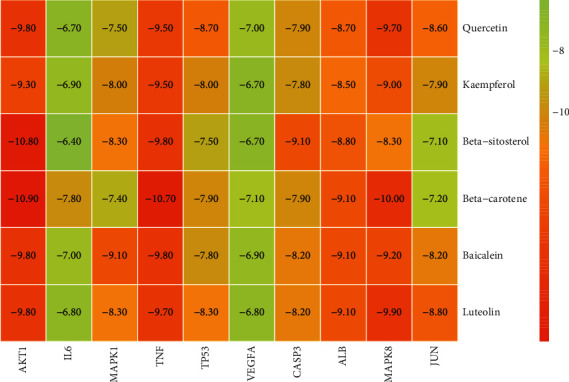
Heatmap of the scores of molecular docking in this study.

**Figure 10:**
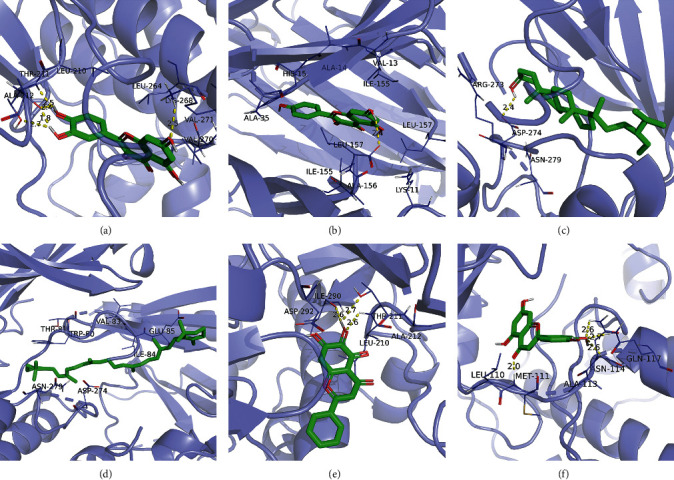
Results of molecular docking between hub genes and key active compounds. (a) Quercetin to AKT1. (b) Kaempferol to TNF. (c) Beta-sitosterol to AKT1. (d) Beta-carotene to AKT1. (e) Baicalein to AKT1. (f) Luteolin to MAPK8.

Sankey diagram was used to establish one-to-one correspondence between the top 10 hub genes (AKT1, IL6, MAPK1, TNF, TP53, VEGFA, CASP3, ALB, MAPK8, and JUN) for BYHWD anti-SCI and BYHWD-related compounds, providing the basis for the next molecular docking analysis. Among them, the gene that targets the most active compounds is TNF, and the key active compounds that target the most hub genes are quercetin and luteolin (Figure 8). As seen in the Sankey diagram, 6 of the 33 active compounds (quercetin, luteolin, beta-carotene, kaempferol, baicalein, and beta-sitosterol) targeted more hub genes, so we decided to perform the molecular docking between these 6 active compounds and top 10 hub genes. The results of the molecular docking score are presented as a heat map (Figure 9), with a range of −6.4 to −10.9 kcal·mol^−1^, representing the large molecule protein receptor (hub genes) that binds well to the small molecule ligand (key active compounds). As shown in Figure 10, the molecular docking process of each key active compound and its corresponding protein encoded by the hub gene with the best docking affinity was visualized using Pymol.  Table 4 shows the basic information of active compounds targeting hub genes in BYHWD.

## 4. Discussion

According to the report of the National SCI Statistics Center, there are about 17,000 new cases of SCI in the United States each year, and the annual incidence of SCI is about 54 cases per million people [1]. Over the past century, advances in understanding the mechanisms of SCI have changed the clinical management strategies for SCI, including surgical procedures, supportive measures, and rehabilitative training that have improved neurological outcomes and reduced morbidity in SCI patients [1]. However, there is still a lack of effective treatments for SCI and thus has been stimulating the desire of researchers to explore and try to find effective treatments, including drugs, surgical methods, and rehabilitation programs. As we all know, TCM has a long history of being used to treat various diseases in China and other Asian countries [28]. Among them, BYHWD, Jisuikang, single herb, and Governor Vessel electroacupuncture have all been shown in relevant studies to be used in the treatment of SCI [4, 5, 28–30].

BYHWD, which comes from Qing Dynasty medical classics, consists of seven herbs: Chishao, Chuanxiong, Danggui, Dilong, Huangqi, Honghua, and Taoren [5]. Based on the theory of TCM, Huangqi is the main ingredient in this formula, which has the function of tonifying Qi and nourishing blood, eliminating blood stasis without harming the proper function; Danggui is the secondary herb, which has the effect of invigorating blood and harmonizing blood; Chishao, Chuanxiong, Dilong, Honghua, and Taoren are the adjuvant herbs in this formula; all the five herbs have the function of activating blood circulation and removing blood stasis, promoting the flow of Qi and blood circulation; the combination of the five herbs not only activates blood circulation and removes blood stasis but also helps the Qi and blood that the main herbs benefit to reach the whole body [3, 31]. Studies have shown that BYHWD in combination with neural stem cells (NSCs) attenuates the process of demyelination or improves the recovery of myelin and exerts a synergistic effect on the neurological function recovery [5]. It has been shown that BYHWD restores hindlimb motor function in rats with SCI, and the neuroprotective effect is associated with the regulation of apoptosis-related protein expression [32]. BYHWD rescues axotomized neurons and promotes functional recovery after spinal cord injury in rats. A study published in 2008 showed that BYHWD protected the injured neurons and promoted functional recovery after SCI in rats [33]. However, since the specific mechanism of BYHWD for the treatment of SCI is still unclear due to its complex composition and wide range of effects, we decided to use a network pharmacology approach to investigate in this study and to lay the foundation for the next in-depth study.

In the first step, BYHWD-related compounds and related targets were obtained. We obtained relevant compounds and targets by mining two herbal-related databases, TCMSP and BATMAN-TCM. The mining methods of these two databases are different: TCMSP database (ADME principle: OB ≥ 30 and DL ≥ 0.18) first obtains BYHWD-related compounds, then obtains related target proteins, and finally needs to be converted into standard gene names by UniProt database; BATMAN-TCM platform (score cutoff >25, adjusted *P* value <0.05) directly obtains BYHWD-related compounds and target genes. The screening process of TCMSP database is divided into five steps: (1) the relevant compounds of each component of BYHWD are directly obtained (Chishao: 119, Chuanxiong: 189, Danggui: 125, Huangqi: 87, Honghua: 189, Taoren: 66, and Dilong: 0); (2) the relevant compounds of each component are screened by ADME criteria (Chishao: 29, Chuanxiong: 7, Danggui: 2, Huangqi: 20, Honghua: 22, Taoren: 23, and Dilong: 0); (3) the above compounds are screened again by removing the null targets (Chishao: 14, Chuanxiong: 6, Danggui: 2, Huangqi: 17, Honghua: 17, Taoren: 19, and Dilong: 0); (4) the above compounds are finally screened by removing duplicates (total number of active compounds: 60); and (5) the target proteins corresponding to the obtained active compounds are converted into gene names (total number of targets: 227). And the screening process of BATMAN-TCM platform is divided into two steps: (1) the relevant compounds and targets of each component of BYHWD are directly obtained (Chishao: 8, Chuanxiong: 87, Danggui: 77, Huangqi: 23, Honghua: 22, Taoren: 2, and Dilong: 0), and (2) the relevant compounds of each component are screened after deduplication (total number of active compounds: 189; the total number of targets: 586). The results of the two databases were integrated and deduplicated to finally obtain 244 BYHWD-related active compounds and 744 BYHWD-related targets. In the second step, a total of 923 SCI-related targets were acquired by mining 5 disease databases, including DisGeNET (number: 84), TTD (number: 5), CTD (number: 553), GeneCards (number: 432), and MalaCards (number: 19). Next, the Venn diagram of BYHWD-related targets and SCI-related targets was plotted by the website online tool to obtain 189 potential therapeutic targets of BYHWD against SCI.

In total, Cytoscape software was used four times in this study: (1) the “BYHWD-active compounds-target genes-SCI” network was constructed, involving 410 nodes and 1571 interactions; (2) the PPI network obtained from the STRING database was reconstructed here, involving 189 nodes and 3717 interactions; (3) the top 10 hub genes (AKT1, IL6, MAPK1, TNF, TP53, VEGFA, CASP3, ALB, MAPK8, and JUN) of BYHWD against SCI were obtained based on 12 topological analysis methods (Degree, MCC, Stress, ClusteringCoefficient, EcCentricity, BottleNeck, Closeness, Radiality, Betweenness, EPC, DMNC, and MNC) contained in the CytoHubba plug-in; (4) the “potential therapeutic targets-pathways” network was constructed, involving 347 nodes and 2650 interactions. Studies have shown that the protein encoded by TP53 and other factors regulate the regeneration, germination, and functional recovery of axons after central nervous system injury [34]; the rapid upregulation of CASP3 mRNA seen after SCI in rats may be associated with cell death in the spinal cord [35]; inflammatory mediators such as TNF-*α* and IL6 mediate the recruitment of inflammatory cells to the site of injury and by targeting these cytokines may be a potential strategy to reduce secondary injury in SCI [36, 37]; by targeting MAPK1, the overexpression of miRNA-433-5p protects motor dysfunction and inflammation after SCI [38].

The Sankey diagram was used once, mainly to show the correspondence between the top 10 hub genes and the corresponding active compounds contained in BYHWD (quercetin, luteolin, beta-carotene, kaempferol, baicalein, beta-sitosterol, carvacrol, O-cresol, anisic acid, 1-methyl-2-dodecyl-4-(1h)-quinolone, 4-ethylresorcinol, angelicin, astragaloside I, astragaloside II, caffeic acid dimethyl ether, cibarian, cordycepin, dihydropinosylvin, dodecenoic acid, ellagic acid, ethyl-P-methoxycinnamate, formononetin, linoleic acid, M-cresol, M-ethylphenol, O-ethylphenol, oleic acid, paeoniflorin, P-cresol, P-ethylphenol, sebiferic acid, sucrose, and thymol). Among them, quercetin targeted six hub genes, luteolin targeted six hub genes, beta-carotene targeted five hub genes, kaempferol targeted four hub genes, baicalein targeted three hub genes, beta-sitosterol targeted two hub genes, anisic acid targeted two hub genes, and the others targeted one hub gene. The experimental results showed that quercetin exerts a protective effect on the spinal cord by inhibiting the activation of the p38MAPK/iNOS signaling pathway in SCI rats and subsequently regulating secondary oxidative stress [39]; the combination of luteolin and palmitoylethanolamide reduced autophagy in SCI [40]; beta-carotene effectively reduced the course of secondary damage events after SCI by blocking NF-*κ*B pathway activation [41]; baicalein may alleviate the harm caused by SCI by activating PI3K and inducing autophagy to reduce neuronal apoptosis [42].

Based on the results of the Sankey diagram, we performed molecular docking of the top 10 genes and the top 6 compounds in terms of the number of targeted genes (quercetin, luteolin, beta-carotene, kaempferol, baicalein, and beta-sitosterol). The results of molecular docking of 60 cohorts of hub target proteins and key active compounds showed a good binding affinity for both. We used R software twice, once to present the scores of the above molecular docking as a heat map, and another time to perform GO and KEGG pathway enrichment analysis using the ClusterProfiler package.

The top 10 results of the GO enrichment analysis of 189 potential therapeutic targets for BYHWD anti-SCI, based on the adjusted *P* value (from small to large), are as follows: response to metal ion (GO-BP:0010038), response to nutrient levels (GO-BP:0031667), response to lipopolysaccharide (GO-BP:0032496), response to molecule of bacterial origin (GO-BP:0002237), cellular response to chemical stress (GO-BP: 0062197), response to antibiotic (GO-BP:0046677), response to oxidative stress (GO-BP: 0006979), response to reactive oxygen species (GO-BP:0000302), response to nutrient (GO-BP:0007584), cellular response to oxidative stress (GO-BP: 0034599), membrane raft (GO-CC:0045121), membrane microdomain (GO-CC:0098857), membrane region (GO-CC:0098589), vesicle lumen (GO-CC: 0031983), protein kinase complex (GO-CC: 1902911), RNA polymerase II transcription regulator complex (GO-CC: 0090575), serine/threonine-protein kinase complex (GO-CC:1902554), secretory granule lumen (GO-CC: 0034774), cytoplasmic vesicle lumen (GO-CC: 0060205), transcription regulator complex (GO-CC: 0005667), RNA polymerase II-specific DNA-binding transcription factor binding (GO-MF:0061629), DNA-binding transcription factor binding (GO-MF:0140297), tetrapyrrole binding (GO-MF:0046906), heme binding (GO-MF:0020037), electron transfer activity (GO-MF: 0009055), nuclear receptor activity (GO-MF:0004879), ligand-activated transcription factor activity (GO-MF:0098531), ubiquitin-like protein ligase binding (GO-MF: 0044389), steroid hormone receptor activity (GO-MF:0003707), and scaffold protein binding (GO-MF:0097110). The first 20 results of the (adjusted *P* value based) KEGG pathway enrichment analysis of 189 potential therapeutic targets of BYHWD against SCI have been presented, and we screened the enriched pathways again to find possible relevant pathways for SCI. By searching the relevant literature, we screened a total of 15 pathways with strong relevance, as follows: AGE-RAGE signaling pathway in diabetic complications (hsa04933) [43], TNF signaling pathway (hsa04668), Apoptosis (hsa04210) [44], Pathways of neurodegeneration-multiple diseases (hsa05022), Toll-like receptor signaling pathway (hsa04620) [45], PI3K-Akt signaling pathway (hsa04151) [46], HIF-1 signaling pathway (hsa04066) [47], MAPK signaling pathway (hsa04010) [48], NF-kappa B signaling pathway (hsa04064) [49], EGFR tyrosine kinase inhibitor resistance (hsa01521) [50], JAK-STAT signaling pathway (hsa04630) [51], Wnt signaling pathway (hsa04310) [43], AMPK signaling pathway (hsa04152) [44], and mTOR signaling pathway (hsa04150) [46]. The above pathways and hub genes are not in a disconnected relationship; rather, many hub genes are important components of some of the pathways. Taking PI3K-Akt signaling pathway as an example, it contains AKT1, MAPK1, IL6, TP53, and VEGFA. Based on the results of the above network pharmacological analysis, we hypothesize that the key active compounds of BYHWD may exert anti-SCI effects through the above pathways and hub genes.

### 4.1. Limitations

This study still has some limitations. First, the corresponding experimental validation is lacking. Second, the compounds, targets, and pathways contained in these databases may not be exhaustive. Finally, compounds screened based on ADME principles may be missing other important compounds.

### 4.2. Future Perspectives

This study uncovered the multicompound, multitarget, and multipathway characteristics of BYHWD through a network pharmacology approach. Among them, the key compounds include quercetin, luteolin, beta-carotene, kaempferol, baicalein, and beta-sitosterol; the hub genes include AKT1, IL6, MAPK1, TNF, TP53, VEGFA, CASP3, ALB, MAPK8, and JUN; and the important pathways are the 15 related pathways mentioned above. These results clearly give a general direction for SCI-related researchers, and there is no doubt that the anti-SCI mechanism of BYHWD is much more than the above-mentioned research directions, and we will find more accurate targets and pathways while exploring them one by one.

## 5. Conclusion

Using a network pharmacology approach, we explored the potential mechanism of BYHWD as a classical herbal formula for treating nerve injury in the treatment of SCI. The top 10 core genes among the potential therapeutic targets for BYHWD anti-SCI include AKT1, IL6, MAPK1, TNF, TP53, VEGFA, CASP3, ALB, MAPK8, and JUN. Key active compounds of BYHWD against SCI include quercetin, luteolin, beta-carotene, kaempferol, baicalein, and beta-sitosterol. In addition, combining the KEGG pathway enrichment results and reviewing SCI-related literature, we obtained 15 SCI potentially relevant pathways, mainly including PI3K-Akt signaling pathway, MAPK signaling pathway, NF-kappa B signaling pathway, TNF signaling pathway, Apoptosis, Toll-like receptor signaling pathway, HIF-1 signaling pathway, JAK-STAT signaling pathway, Wnt signaling pathway, AMPK signaling pathway, and mTOR signaling pathway. The above analysis results demonstrated the multitarget, multicomponent, and multipathway characteristics of BYHWD in treating SCI, which laid the foundation for us to carry out specific experimental validation in the next step, and provided new ideas for researchers dedicated to the treatment of SCI with herbal extracts.

## Figures and Tables

**Table 1:** Basic information of hub genes based on 12 topological algorithms.

UniProt ID	Gene symbol	Protein names	Algorithms
P31749	AKT1	RAC-alpha serine/threonine-protein kinase	(1), (2), (3), (4), (5), (8), (10), (11), and (12)
P05231	IL6	Interleukin-6	(1), (2), (3), (4), (5), (8), (10), (11), and (12)
P28482	MAPK1	Mitogen-activated protein kinase 1	(1), (2), (3), (5), (8), (9), (10), (11), and (12)
P01375	TNF	Tumor necrosis factor	(1), (2), (3), (4), (5), (8), (10), (11), and (12)
P04637	TP53	Cellular tumor antigen p53	(1), (2), (3), (4), (5), (8), (10), (11), and (12)
P15692	VEGFA	Vascular endothelial growth factor A	(1), (2), (3), (4), (5), (8), (10), (11), and (12)
P42574	CASP3	Caspase-3	(1), (2), (3), (5), (8), (10), (11), and (12)
P02768	ALB	Albumin	(1), (3), (5), (8), (10), (11), and (12)
P45983	MAPK8	Mitogen-activated protein kinase 8	(1), (2), (4), (5), (8), and (10)
P05412	JUN	Transcription factor AP-1	(1), (2), (5), (10), and (11)
P04040	CAT	Catalase	(3), (4), and (12)
P99999	CYCS	Cytochrome c	(3) and (12)
P09238	MMP10	Stromelysin-2	(6) and (7)
P07101	TH	Tyrosine 3-monooxygenase	(4) and (9)
P37023	ACVRL1	Serine/threonine-protein kinase receptor R3	(6)
P01019	AGT	Angiotensinogen	(9)
P09917	ALOX5	Polyunsaturated fatty acid 5-lipoxygenase	(6)
P01185	AVP	Vasopressin-neurophysin 2-copeptin	(9)
Q07812	BAX	Apoptosis regulator BAX	(6)
O15392	BIRC5	Baculoviral IAP repeat-containing protein 5	(6)
P55211	CASP9	Caspase-9	(7)
P20248	CCNA2	Cyclin-A2	(7)
P14635	CCNB1	G2/mitotic-specific cyclin-B1	(7)
P13073	COX4I1	Cytochrome c oxidase subunit 4 isoform 1, mitochondrial	(9)
P02741	CRP	C-reactive protein	(4)
P02778	CXCL10	C-X-C motif chemokine 10	(7)
P00533	EGFR	Epidermal growth factor receptor	(11)
P15407	FOSL1	Fos-related antigen 1	(6)
P15408	FOSL2	Fos-related antigen 2	(6)
Q13224	GRIN2B	Glutamate receptor ionotropic, NMDA 2B	(9)
Q16665	HIF1A	Hypoxia-inducible factor 1-alpha	(9)
P04792	HSPB1	Heat shock protein beta-1	(7)
P05362	ICAM1	Intercellular adhesion molecule 1	(7)
P35225	IL13	Interleukin-13	(7)
P05112	IL4	Interleukin-4	(7)
P01374	LTA	Lymphotoxin-alpha	(6)
P01106	MYC	Myc proto-oncogene protein	(8)
P29474	NOS3	Nitric oxide synthase, endothelial	(9)
O14684	PTGES	Prostaglandin E synthase	(6)
P35354	PTGS2	Prostaglandin G/H synthase 2	(2)
Q15858	SCN9A	Sodium channel protein type 9 subunit alpha	(4)
P16581	SELE	E-selectin	(7)
P07204	THBD	Thrombomodulin	(6)
Q8NER1	TRPV1	Transient receptor potential cation channel subfamily V member 1	(9)
P11473	VDR	Vitamin D3 receptor	(9)

(1): Degree, (2): Maximal Clique Centrality (MCC), (3): Betweenness, (4): BottleNeck, (5): Closeness, (6): ClusteringCoefficient, (7): Density of Maximum Neighborhood Component (DMNC), (8): Edge Percolated Component (EPC), (9): EcCentricity, (10): Maximum Neighborhood Component (MNC), (11): Radiality, and (12): Stress

**Table 2:** Top 10 items of Gene Ontology (GO) enrichment analysis.

GO items	ID	Description	*P* value	Adjusted *P* value	Gene number
Biological process	GO:0010038	Response to metal ion	2.68739*E*−45	1.20529*E*−41	52
Biological process	GO:0031667	Response to nutrient levels	2.05974*E*−40	4.61896*E*−37	54
Biological process	GO:0032496	Response to lipopolysaccharide	5.34106*E*−38	7.98488*E*−35	45
Biological process	GO:0002237	Response to molecule of bacterial origin	3.1151*E*−37	3.4928*E*−34	45
Biological process	GO:0062197	Cellular response to chemical stress	1.44121*E*−35	1.29277*E*−32	44
Biological process	GO:0046677	Response to antibiotic	2.54411*E*−34	1.90172*E*−31	42
Biological process	GO:0006979	Response to oxidative stress	3.05284*E*−34	1.956*E*−31	47
Biological process	GO:0000302	Response to reactive oxygen species	9.13447*E*−34	5.12101*E*−31	37
Biological process	GO:0007584	Response to nutrient	2.40193*E*−33	1.19696*E*−30	36
Biological process	GO:0034599	Cellular response to oxidative stress	6.07803*E*−32	2.726*E*−29	39
Cellular component	GO:0045121	Membrane raft	3.92204*E*−13	7.60946*E*−11	22
Cellular component	GO:0098857	Membrane microdomain	4.18102*E*−13	7.60946*E*−11	22
Cellular component	GO:0098589	Membrane region	8.84519*E*−13	1.07322*E*−10	22
Cellular component	GO:0031983	Vesicle lumen	4.00266*E*−10	3.64242*E*−08	19
Cellular component	GO:1902911	Protein kinase complex	5.90811*E*−10	4.10759*E*−08	12
Cellular component	GO:0090575	RNA polymerase II transcription regulator complex	7.53142*E*−10	4.10759*E*−08	14
Cellular component	GO:1902554	Serine/threonine-protein kinase complex	7.89922*E*−10	4.10759*E*−08	11
Cellular component	GO:0034774	Secretory granule lumen	2.08462*E*−09	9.48503*E*−08	18
Cellular component	GO:0060205	Cytoplasmic vesicle lumen	2.53536*E*−09	1.02541*E*−07	18
Cellular component	GO:0005667	Transcription regulator complex	3.48128*E*−09	1.26718*E*−07	20
Molecular function	GO:0061629	RNA polymerase II-specific DNA-binding transcription factor binding	1.80793*E*−11	7.91763*E*−09	20
Molecular function	GO:0140297	DNA-binding transcription factor binding	2.70226*E*−11	7.91763*E*−09	22
Molecular function	GO:0046906	Tetrapyrrole binding	4.34881*E*−11	8.49468*E*−09	15
Molecular function	GO:0020037	Heme binding	1.91194*E*−10	2.801*E*−08	14
Molecular function	GO:0009055	Electron transfer activity	2.62858*E*−10	3.08069*E*−08	13
Molecular function	GO:0004879	Nuclear receptor activity	1.43492*E*−09	1.20123*E*−07	9
Molecular function	GO:0098531	Ligand-activated transcription factor activity	1.43492*E*−09	1.20123*E*−07	9
Molecular function	GO:0044389	Ubiquitin-like protein ligase binding	5.74415*E*−09	4.20759*E*−07	18
Molecular function	GO:0003707	Steroid hormone receptor activity	7.34487*E*−09	4.78232*E*−07	9
Molecular function	GO:0097110	Scaffold protein binding	1.18514*E*−08	5.67261*E*−07	9

**Table 3:** The enriched 15 possible related pathways for SCI.

ID	Description	*P* value	Adjusted *P* value	Gene number
hsa04933	AGE-RAGE signaling pathway in diabetic complications	8.58479*E*−34	2.24063*E*−31	35
hsa04668	TNF signaling pathway	8.83261*E*−24	3.84219*E*−22	29
hsa04210	Apoptosis	1.13129*E*−15	1.42828*E*−14	24
hsa05022	Pathways of neurodegeneration - multiple diseases	1.12387*E*−14	1.0476*E*−13	41
hsa04620	Toll-like receptor signaling pathway	5.71758*E*−14	4.52208*E*−13	20
hsa04151	PI3K-Akt signaling pathway	1.43931*E*−13	1.00595*E*−12	34
hsa04066	HIF-1 signaling pathway	1.4646*E*−13	1.00595*E*−12	20
hsa04010	MAPK signaling pathway	5.84978*E*−12	3.39287*E*−11	29
hsa04064	NF-kappa B signaling pathway	7.32616*E*−12	4.1568*E*−11	18
hsa01521	EGFR tyrosine kinase inhibitor resistance	1.25843*E*−09	5.13202*E*−09	14
hsa04012	ErbB signaling pathway	3.21508*E*−08	1.0622*E*−07	13
hsa04630	JAK-STAT signaling pathway	4.40433*E*−07	1.27726*E*−06	16
hsa04310	Wnt signaling pathway	0.000748937	0.001515292	11
hsa04152	AMPK signaling pathway	0.001230137	0.002407294	9
hsa04150	mTOR signaling pathway	0.002091493	0.003927191	10

**Table 4:** Basic information of active compounds targeting hub genes in BYHWD.

Molecule ID	Molecule name	PubChem CID	OB (%)	DL	2D Structure	Source database	Source	Targeted hub genes
MOL000098	Quercetin	5280343	46.43	0.28	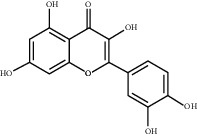	TCMSP	Honghua; Huangqi	AKT1, CASP3, IL6, JUN, MAPK1, and VEGFA
MOL000006	Luteolin	5280445	36.16	0.25	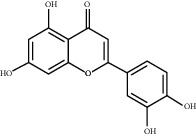	TCMSP	Honghua	AKT1, CASP3, IL6, JUN, MAPK1, and VEGFA
MOL002773	Beta-carotene	5280489	37.18	0.58	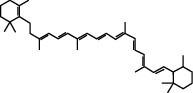	TCMSP	Honghua	AKT1, CASP3, ALB, JUN, and VEGFA
MOL000422	Kaempferol	5280863	41.88	0.24	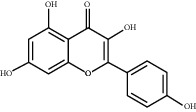	TCMSP	Honghua; Huangqi	AKT1, CASP3, JUN, and MAPK8
MOL002714	Baicalein	5281605	33.52	0.21	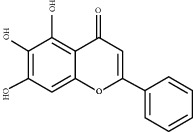	TCMSP	Chishao; Honghua	AKT1, CASP3, and VEGFA
MOL000358	Beta-sitosterol	222284	36.91	0.75	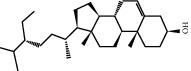	TCMSP	Chishao, Danggui, Honghua, and Taoren	CASP3 and JUN
MOL001002	Ellagic acid	5281855	43.06	0.43	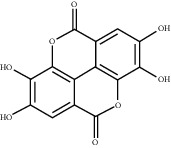	TCMSP	Chishao	VEGFA
MOL001924	Paeoniflorin	442534	53.87	0.79	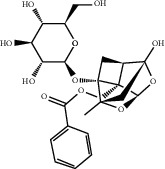	TCMSP	Chishao	IL6
MOL000392	Formononetin	5280378	69.67	0.21	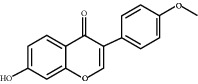	TCMSP	Huangqi	JUN
—	Anisic acid	7478	—	—	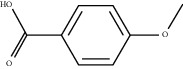	BATMAN-TCM	Danggui	TNF and TP53
—	Caffeic acid dimethyl ether	717531	—	—	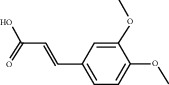	BATMAN-TCM	Chuanxiong	TNF
—	Cibarian	100275	—	—	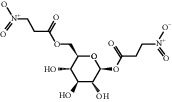	BATMAN-TCM	Chuanxiong	ALB
—	Linoleic acid	5280450	—	—	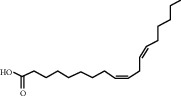	BATMAN-TCM	Chuanxiong	TNF
—	O-Cresol	335	—	—	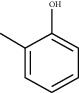	BATMAN-TCM	Chuanxiong	TNF
—	O-Ethylphenol	6997	—	—	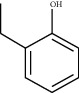	BATMAN-TCM	Chuanxiong	TNF
—	Oleic acid	445639	—	—	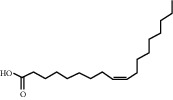	BATMAN-TCM	Chuanxiong	TNF
—	Thymol	6989	—	—	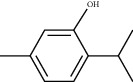	BATMAN-TCM	Chuanxiong	TNF
—	1-Methyl-2-dodecyl-4-(1h)-quinolone	5319601	—	—	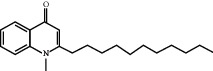	BATMAN-TCM	Danggui	JUN
—	4-Ethylresorcinol	17927	—	—	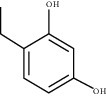	BATMAN-TCM	Danggui	TNF
—	Angelicin	10658	—	—	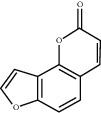	BATMAN-TCM	Danggui	AKT1
—	Carvacrol	10364	—	—	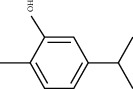	BATMAN-TCM	Danggui	TNF
—	Dihydropinosylvin	442700	—	—	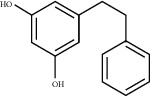	BATMAN-TCM	Danggui	TNF
—	Dodecenoic acid	96204	—	—	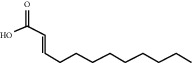	BATMAN-TCM	Danggui	TNF
—	Ethyl-P-methoxycinnamate	5281783	—	—	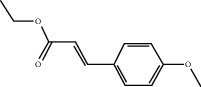	BATMAN-TCM	Danggui	TNF
—	M-Cresol	342	—	—	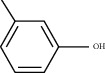	BATMAN-TCM	Danggui	TNF
—	M-Ethylphenol	12101	—	—	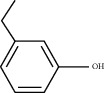	BATMAN-TCM	Danggui	TNF
—	P-Cresol	2879	—	—	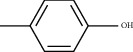	BATMAN-TCM	Danggui	TNF
—	P-Ethylphenol	31242	—	—	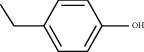	BATMAN-TCM	Danggui	TNF
—	Sebiferic acid	5321206	—	—	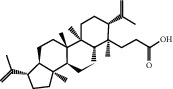	BATMAN-TCM	Danggui	TNF
—	Astragaloside I	13996685	—	—	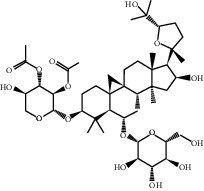	BATMAN-TCM	Huangqi	ALB
—	Astragaloside II	13996693	—	—	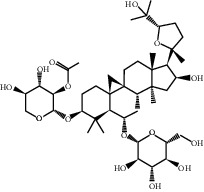	BATMAN-TCM	Huangqi	ALB
—	Sucrose	5988	—	—	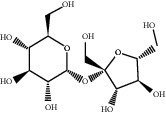	BATMAN-TCM	Huangqi	TNF
—	Cordycepin	6303	—	—	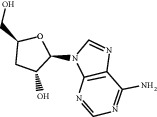	BATMAN-TCM	Taoren	AKT1

OB, oral bioavailability; DL, drug-likeness.

## Data Availability

The data used to support the findings of this study are included within the article.
